# The development of temporal cognition in hearing-impaired children and educational recommendations

**DOI:** 10.3389/fpsyg.2025.1623713

**Published:** 2025-10-01

**Authors:** Yanhong Fang, Guanhai Yin

**Affiliations:** ^1^Faculty of Education, Guangxi Normal University, Guilin, Guangxi, China; ^2^School of Education, Jinggangshan University, Ji'an, Jiangxi, China

**Keywords:** hearing-impaired children, temporal succession cognition, developmental trajectories, compensatory education, temporal cognition

## Abstract

An experiment was conducted by temporal sequencing, selection, and judgment tasks to investigate the developmental characteristics of temporal cognition in hearing-impaired children and propose targeted educational strategies. Participants included 79 hearing-impaired students (aged 11–17) from special education schools, grouped into lower- (11–13 years), middle- (14–15 years) and upper-grade (16–17 years) cohorts. All participants had bilateral sensorineural hearing loss, used hearing aids regularly, and received structured auditory rehabilitation. Tasks assessed both concrete short-term sequences (e.g., water flowing in funnels) and integrated long-term sequences (e.g., seasonal cycles, abstract time concepts). Results revealed three key findings including: (1) Age-Related Progression. Temporal cognition in hearing-impaired children exhibited a clear developmental trajectory, with performance improving steadily with age. A rapid growth phase was observed in the middle-grade group (14–15 years), indicating a critical period for intervention; (2) Concrete vs. Integrated Temporal Cognition. Children demonstrated superior performance in short-term, concrete temporal tasks (e.g., daily routines) compared to long-term, integrated tasks (e.g., abstract time concepts or seasonal cycles); (3) Future-Oriented Bias: Future-oriented temporal cognition significantly outperformed past-oriented cognition, suggesting unique compensatory mechanisms in temporal reasoning. Time span, temporal representation, children’s life experiences, and event complexity were important factors that affected the temporal cognitive development of hearing-impaired children. These findings underscore the need for tailored educational approaches that leverage visual–spatial strengths and experiential learning and inform auditory rehabilitation practices.

## 1 Introduction

Temporal cognition constitutes a fundamental aspect of human cognitive development, encompassing the ability to perceive and interpret the continuity, sequentiality, and duration of events. It consists of two interdependent dimensions: temporal succession (the ordering of events) and duration estimation (the perception of intervals), which together form its foundational bedrock.

The development of this capacity is critical not only for children’s understanding of the physical and social world but also serves as a psychological prerequisite for knowledge acquisition across disciplines. It enables children to anticipate future events, plan actions, and reflect on past experiences—skills essential for adaptive functioning and intellectual growth (Liang, 2002; Fang, 2001). Given its centrality, psychologists and educators have long sought to unravel the mechanisms and developmental trajectories of temporal cognition. Furthermore, temporal processing is a core component of central auditory processing, directly influencing—and often being assessed in diagnoses of auditory processing disorders (CAPD)—speech perception, language acquisition, and literacy skills (American Academy of Audiology, 2010). Over the past century, a rich body of research has employed diverse methodologies to explore how children conceptualize time across different developmental stages.

### 1.1 Milestones in temporal succession understanding

This development is supported by underlying neurological and auditory maturation, which facilitates the processing of increasingly rapid and complex acoustic cues.

Seminal work by Friedman (2002) utilized temporal sequencing tasks to investigate children’s ability to organize daily routines. This research revealed that early temporal cognition is rooted in familiar, repetitive experiences. Friedman further explored children’s grasp of temporal lag, demonstrating age-related improvements in estimating distances between future events. These behavioral advancements are underpinned by progressive neurological maturation. The development of temporal sequencing abilities is closely linked to the maturation of prefrontal cortex (PFC) and medial temporal lobe structures, which support working memory, executive function, and long-term memory integration—all essential for manipulating temporal concepts (Pathman et al., 2018). Similarly, auditory maturation, particularly in the central auditory pathways, enhances the ability to process rapid temporal acoustic cues crucial for speech perception and the development of precise internal timing mechanisms (Moore and Linthicum, 2007).

Benson (1997) expanded this line of inquiry by examining children’s knowledge of routine sequences associated with “yesterday,” “today,” and “tomorrow.” Their findings highlighted an age-dependent progression in sequential reasoning. The research of Liang’s (2002) offered a contrasting perspective, demonstrating that even young children could distinguish and sequence future-oriented “tomorrow” events with surprising accuracy.

Further insights emerged from studies investigating children’s comprehension of cyclical timeframes. Fang et al. (1984a, 1984b) revealed that shorter cycles (e.g., “a day”) were mastered earlier than longer ones (e.g., “a week”). This proximal-to-distal trajectory is further illustrated in children’s understanding of large-scale, natural cycles. Seminal work by Friedman (1977, 1986) employed tasks such as sequencing the four seasons to demonstrate how children’s grasp of long-term, periodic time develops from concrete experiences (e.g., snow for winter) to an abstract, logical order throughout middle childhood. This aligns with the broader principle that temporal cognition is guided by both experiential learning and the ongoing neural specialization of relevant brain networks (Pathman et al., 2023; Samaritzi, 2011; Pathman et al., 2018).

Synthesizing these findings, researchers have delineated three developmental phases:

Emergent Phase (0–3 years): Infants and toddlers exhibit nascent sensitivity to temporal patterns through rhythmic interactions and sensorimotor experiences.

Developmental Phase (4–7 years): Children begin to construct explicit temporal frameworks, sequencing familiar events and using basic temporal terms. This phase coincides with significant synaptic pruning and myelination in language-associated cortical areas.

Refinement Phase (8 + years): Abstract temporal reasoning emerges, enabling children to manipulate complex sequences and integrate multiple temporal reference points, supported by more adult-like functioning of the fronto-parietal network.

### 1.2 The challenge of duration perception

Piaget’s pioneering studies laid the groundwork for understanding children’s duration perception. He posited that children initially conflate time with spatial or intensive properties (e.g., speed, distance) and only later develop pure temporal understanding (Piaget, 1987). Subsequent research has complicated this narrative, showing that children’s duration judgments are influenced by various perceptual factors (Huang and Zheng, 1993). These findings underscore the multisensory and context-dependent nature of early temporal processing.

Longitudinal investigations further revealed age-related improvements in duration perception. By age 8, children begin to utilize internal “timekeepers” to gauge intervals, though their estimates remain variable. Mastery of duration calculation emerges around ages 11–12, while the ability to discount extraneous factors stabilizes only in early adolescence (Huang and Zheng, 1993).

### 1.3 Temporal cognition in hearing-impaired children: gaps and implications

While extensive research has mapped temporal cognition in typically developing children, far less is known about hearing-impaired populations. Hearing impairment is a heterogeneous condition with varying etiologies (genetic, acquired), degrees (mild to profound), and configurations. This heterogeneity can lead to differential impacts on cognitive development, particularly in domains requiring precise temporal processing (Brown, 2019). Congenital hearing loss disrupts the typical auditory experience that is fundamental for tuning the neural encoding of temporal patterns in the environment. This disruption is not merely sensory but has cascading effects on neurocognitive development.

Early work by Neville and colleagues demonstrated that auditory deprivation triggers cross-modal plasticity, where auditory cortices are recruited for visual and somatosensory processing (Neville and Bavelier, 2001). While this can enhance visual temporal resolution in some tasks (e.g., motion detection in the periphery), it can also lead to deficits in tasks requiring millisecond-level audiovisual temporal precision (e.g., speech reading, synchrony detection) (Hauthal et al., 2015). This suggests a trade-off between sensory compensation and the development of supramodal temporal integration mechanisms.

Zhang’s (2006) seminal work revealed that auditory deprivation profoundly impacts higher-order temporal processing. Deaf children exhibit deficits in sequential memory and complex duration estimation, which are critical for language comprehension and academic learning. Chen et al. (2017) found that deaf and hard-of-hearing children had significantly longer thresholds for detecting visual sequence changes compared to hearing peers, suggesting reduced efficiency in rapid temporal binding. These challenges are compounded by language delays, which limit exposure to and practice with temporal vocabulary (e.g., before, after, next, last year) and narrative structures that reinforce temporal concepts.

Despite these insights, critical questions remain unanswered regarding how hearing-impaired children with varied audiological profiles process complex temporal sequences and conceptualize integrated time across different domains. Addressing these gaps holds profound implications for special education, as temporal cognition underpins academic skills such as reading comprehension (following narrative sequences), mathematics (grasping time-related problems), and science (understanding cyclical processes). For hearing-impaired children, who already face linguistic and social barriers, undiagnosed temporal cognition deficits could exacerbate learning challenges. Tailored educational strategies—such as leveraging visual timelines, kinesthetic activities, or technology-assisted timekeeping tools—might mitigate these effects, fostering compensatory pathways for temporal understanding.

This study seeks to illuminate the developmental trajectory of temporal cognition in hearing-impaired children, with dual aims: to delineate how the nature and degree of auditory deprivation shapes the acquisition of succession and duration concepts; and to propose evidence-based interventions that align with their unique neurocognitive profiles.

## 2 Research methodology

### 2.1 Participants

This cross-sectional study was approved by the Institutional Review Board of Guangxi Normal University. All procedures were conducted in accordance with the ethical standards of the responsible committee and with the Helsinki Declaration of 1975, as revised in 2008.

A total of 91 hearing-impaired children from several special education schools in Guangxi Province were initially recruited. However, only 79 participants (45 males, 34 females) met inclusion criteria and were able to comprehend and complete the experimental tasks. Inclusion criteria included: bilateral sensorineural hearing loss, no additional disabilities, normal or corrected-to-normal vision, and ability to complete the experimental tasks. Exclusion criteria included: conductive or mixed hearing loss, cognitive impairment, or visual impairment uncorrected by lenses.

The participants were divided into three age groups:

Lower-grade group: 18 children (aged 11–13 years).

Middle-grade group: 30 children (aged 14–15 years).

Upper-grade group: 31 children (aged 16–17 years).

All participants had bilateral sensorineural hearing loss. Based on their most recent audiograms, the degree of hearing loss was classified as moderate (41–70 dB HL) in 22 children, severe (71–90 dB HL) in 35 children, and profound (>91 dB HL) in 22 children. The majority (86.1%) had congenital non-syndromic hearing loss; the remainder acquired hearing loss before age 3 due to meningitis or ototoxic drug exposure. All children used digitally programmed behind-the-ear (BTE) or in-the-ear (ITE) hearing aids, fitted and validated according to DSL v5.0 targets. The average duration of hearing aid use was 9.3 ± 3.2 years, with average daily use of 8.2 ± 2.1 h. Hearing aid fittings were updated annually or as needed. Children with profound loss who did not benefit sufficiently from hearing aids were referred for cochlear implant evaluation; however, none had received implants at the time of the study. All children attended special education schools and had received structured auditory-speech rehabilitation for at least 5 years. Speech discrimination scores ranged from 40 to 85% in quiet conditions (Table 1).

**Table 1:** Demographic and audiological characteristics of participants.

Characteristic	Lower-grade (*n* = 18)	Middle-grade (*n* = 30)	Upper-grade (*n* = 31)	Total (*n* = 79)
Age (years)	12.2 ± 0.8	14.6 ± 0.5	16.8 ± 0.7	14.9 ± 2.1
Male/Female	10/8	17/13	18/13	45/34
Hearing loss degree				
Moderate	6	9	7	22
Severe	8	13	14	35
Profound	4	8	10	22
Hearing aid use (years)	8.1 ± 2.8	9.5 ± 3.1	10.1 ± 3.5	9.3 ± 3.2
Daily device use (hours)	7.8 ± 2.3	8.3 ± 2.0	8.4 ± 2.1	8.2 ± 2.1

### 2.2 Materials

Seven sets of pictorial materials were designed. Several were adapted from established paradigms in temporal cognition research to ensure validity, while others were created by the authors to address culturally and developmentally relevant long-term sequences. All materials were pilot-tested with 10 hearing-impaired children not participating in the main study to ensure appropriateness and comprehensibility. The seven sets were as follows:

*Daily routines* (e.g., Morning wake-up, school attendance, flag-raising ceremony, recess rope-jumping, evening homework): This set was adapted from the paradigms used by Friedman (2002) to assess sequencing of familiar events. The specific activities were modified to reflect the common daily experiences of children in Chinese special education schools.

*Four seasons* (Spring, summer, autumn, winter): adapted from Friedman’s widely referenced seminal classic (1977, 1986). The images depicting seasonal characteristics (e.g., snow for winter, blooming flowers for spring) were culturally adjusted to represent typical Guangxi seasons.

*Lotus growth stages* (Bud, partial bloom, full bloom, wilting, seedpod). The paradigm of sequencing a plant’s growth was inspired by Piagetian seriation tasks (Piaget and Inhelder, 1969). However, the specific use of the lotus flower was designed by the authors. The lotus was chosen for its distinct, visually salient stages of change and its high cultural recognition within China, which minimizes experimental error due to cultural unfamiliarity.

*Abstract temporal terms* (vocabulary cards for “last year” “yesterday” “today” “next month” “next year”): the methodology for assessing understanding of these terms was developed based on Benson (1997) and Fang et al. (1984a, 1984b). The terms were presented in written Chinese characters.

*Traditional festivals* (Spring Festival, Lantern Festival, Qingming Festival, Dragon Boat Festival and Mid-Autumn Festival): This set was developed by the authors, inspired by Friedman’s (2002) methodology of sequencing annual events. The festival names were replaced with major Chinese festivals to align with the participants’ cultural experiences and assess comprehension of culturally significant annual event sequences.

*Human life stages* (Infant, child, youth, middle-aged adult, elderly): This set was adapted from Friedman (2000) and Titone (1980) who used the human life cycle to investigate cognitive development. The images were selected to be clear and representative of each life stage.

*Water flow in glass funnel* (Sequential images showing 100, 75, 50, 25, and 0% water levels): This task was adapted from the classic Piagetian water pouring paradigm (Piaget, 1987), translated into a static visual sequencing task using clear pictorial cues.

### 2.3 Experimental tasks and procedures

Testing was conducted individually in a quiet room at the participants’ schools. The child was seated approximately 50 cm from a laptop screen. The examiner provided instructions using simultaneous communication (speech and sign). The tasks were administered in a fixed order. Breaks were offered to avoid fatigue. The total duration was approximately 40 min per child.

These tasks were selected based on their established use in prior research with similar populations to ensure comprehensibility and valid participation (Friedman, 1977, 1986, 2000, 2002; Benson, 1997; Fang et al., 1984a, 1984b; Piaget, 1987, etc.), though the potential for ceiling effects on the simpler tasks is acknowledged.

The experiment comprised two main components: concrete short-term temporal cognition and integrated long-term temporal cognition, each involving three subtasks: temporal sequencing, temporal selection and judgment. Performance was evaluated using accuracy rate metric with partial credit scoring to better capture developmental differences.

#### 2.3.1 Concrete Short-Term Temporal Cognition Tasks

##### 2.3.1.1 Sequencing Task (modified Piagetian water flow paradigm)

Children watched the pre-recorded video of water flowing from the separatory funnel to the conical flask. Five still images were captured at 100, 75, 50, 25, and 0% water levels in the upper funnel. These images were scrambled and presented to participants, who were asked to reconstruct the correct sequence. Scoring: 1 point for fully correct sequences, 0.5 points for sequences with one error, and 0 points for more than one error.

##### 2.3.1.2 Selection task

A randomly chosen image (from positions 2, 3, or 4 in the sequence) was presented. Children were asked to select the images that occurred before and after the target. Scoring: 1 point per correct selection (past/future), total = 2 points.

##### 2.3.1.3 Judgment Task

Two images were displayed in either correct (“past → future”) or reversed (“future → past”) order. Children judged the correctness and verbally explained the proper sequence. Scoring: 1 point for correct judgment + explanation; otherwise = 0. Total = 2 points.

#### 2.3.2 Integrated long-term temporal cognition tasks

##### 2.3.2.1 Sequencing task

Children arranged six sets of images/cards into chronological order: daily routines, four seasons, lotus growth, traditional festivals, human life stages, and abstract temporal terms (e.g., words). Scoring:1 point for fully correct sequences, 0.5 points for sequences with one error, and 0 points for more than one error per set (total = 6).

##### 2.3.2.2 Selection task

For each of the six sets, a middle image was randomly selected. Children identified the preceding (“past”) and succeeding (“future”) images. Scoring: 1 point per correct selection (total = 12).

##### 2.3.2.3 Judgment task

Six pre-arranged sequences (e.g., “morning wake-up → school → flag-raising → evening homework → recess”; “yesterday → last year → today → next year → next month”) were presented, some with intentional errors. Children judged their correctness. Scoring: 1 point per correct judgment (total = 6).

### 2.4 Statistical analysis

Data were processed and analyzed using SPSS 23.0. Descriptive statistics, one-way ANOVA was used to compare performance across age groups. When significant main effects were found, Tukey’s HSD post-hoc test was applied to examine pairwise differences. To address the potential confounding effect of hearing loss level, a series of one-way Analyses of Covariance (ANCOVAs) were conducted with Grade as a fixed factor and Hearing Loss Level as a covariate. Significance was set at *p* < 0.05.

## 3 Results and analysis

### 3.1 Performance on concrete short-term temporal cognition tasks

The scores of lower-, middle-, and upper-grade hearing-impaired children on sequencing, selection, and judgment tasks are summarized in Table 2.

**Table 2:** Mean scores and standard deviations across three concrete temporal tasks.

Grade	Sequencing (Mean *± SD*)	Selection (Mean *± SD*)	Judgment (Mean *± SD*)
Lower-grade	0.94 ± 0.24	1.83 ± 0.45	1.83 ± 0.52
Middle-grade	0.97 ± 0.18	1.83 ± 0.34	1.93 ± 0.29
Upper-grade	1.00 ± 0.00	2.00 ± 0.00	2.00 ± 0.00

As shown in Table 2, all age groups achieved perfect scores on the sequencing task (reordering water flow images). Performance on selection and judgment tasks improved with age, with upper-grade children attaining full accuracy. However, statistical tests (ANOVA) revealed no significant intergroup differences in selection, *F*_(2,76)_ = 2.98, *p* > 0.05 or judgment *F*_(2,76)_ = 1.58, *p* > 0.05 scores. These results suggest that hearing-impaired children demonstrate strong competence in perceiving concrete, observable, short-duration temporal sequences, accurately reconstructing event order, processes, and outcomes.

### 3.2 Performance on integrated long-term temporal cognition tasks

Table 3 presents the mean scores for sequencing six integrated temporal categories. According to Table 3, hearing-impaired children showed much lower performance on integrated temporal sequences compared to concrete sequences. The total scores were significantly lower than the maximum possible (6 points), with only 2.83, 3.67, and 4.06 points for lower, middle, and upper-grades, respectively. Among the six time series, children showed the best understanding of daily routines (100% accuracy), followed by human life stages and seasonal changes. This pattern of performance—where concrete, experience-based sequences are mastered before more abstract, culturally-transmitted cycles—echoes the developmental trajectory established in typically developing children (Friedman, 1977). However, they demonstrated poor understanding of traditional festivals, abstract time concepts, and lotus growth processes—events that span longer time periods and are relatively abstract or less frequently experienced. Therefore, the cognitive ability of hearing-impaired children towards different time periods is uneven, and their developmental level is influenced by time intervals, temporal performance attributes, and children’s life experiences. Furthermore, some hearing-impaired children in middle and upper grades rank their daily lives beyond the boundaries of a day, and their ranking of the four seasons of the year also exceeds the boundaries of a year, indicating that the level of temporal cognition is indeed related to children’s experiences. Children use their own life experiences as a reference for understanding temporal relationships, including cyclical lifestyle habits and natural human growth. This pattern, wherein adolescents with hearing loss perform at near-ceiling levels on concrete tasks but show delayed development on abstract ones, highlights a specific profile of cognitive development shaped by auditory experience.

**Table 3:** Mean scores and standard deviations for integrated temporal sequencing.

Grade	Daily routines	Four seasons	Lotus growth	Abstract time	Festivals	Human life	Total score
Lower-grade	1.00 ± 0.00	0.78 ± 0.43	0.00 ± 0.00	0.17 ± 0.38	0.11 ± 0.32	0.78 ± 0.43	2.83 ± 0.38
Middle-grade	1.00 ± 0.00	0.87 ± 0.35	0.23 ± 0.43	0.27 ± 0.45	0.30 ± 0.47	1.00 ± 0.00	3.67 ± 0.88
Upper-grade	1.00 ± 0.00	0.97 ± 0.18	0.26 ± 0.44	0.38 ± 0.49	0.45 ± 0.51	1.00 ± 0.00	4.06 ± 0.67

The ANOVA showed significant differences between grades [*F*_(2,76)_ = 16.86, *p* < 0.001]. Post-hoc tests using Tukey’s HSD revealed that the middle-grade group performed significantly better than the lower-grade group (*p* < 0.001), and the upper-grade group performed significantly better than both lower (*p* < 0.001) and middle (*p* = 0.032) groups. This indicates that temporal sequencing ability continues to develop through adolescence in hearing-impaired children.

### 3.3 Performance on selection and judgment tasks

Table 4 details performance on past/future selection and sequence judgment tasks of different grades on comprehensive time series.

**Table 4:** Scores for integrated temporal selection and judgment tasks.

Grade	Past selection (Mean ± *SD*)	Future selection (Mean ± *SD*)	Total selection (Mean ± *SD*)	Total judgment (Mean ± *SD*)
Lower-grade	3.22 ± 1.66	3.61 ± 1.19	6.83 ± 1.42	3.30 ± 1.84
Middle-grade	4.00 ± 1.11	4.27 ± 1.20	8.27 ± 1.74	4.27 ± 0.98
Upper-grade	4.19 ± 0.94	4.35 ± 1.11	8.54 ± 1.63	4.26 ± 1.15

Due to the different themes of the six groups of pictures and the relatively long time interval represented by each group of pictures, the selection task involves two questions that examine the cognitive abilities of hearing-impaired children in the past and future. Table 4 shows that hearing-impaired children in each age group performed better on future-oriented tasks than past-oriented tasks. A significant difference test was conducted on the average scores of time sequence selection among the three groups. The results showed significant grade differences, *F*_(2,76)_ = 6.72, *p* = 0.002. Multiple comparison analysis showed that the selection performance of middle and upper-grade children was significantly better than that of lower-grade children (*p* = 0.004 and *p* = 0.001 respectively), while there was no significant difference between middle and upper-grade children (*p* = 0.50).

The overall performance on temporal judgment tasks also improved with grade, and ANOVA showed significant grade differences, *F*_(2,76)_ = 3.62, *p* = 0.031. Further analysis showed that although there was no significant difference between middle and upper grades (*p* = 0.50), both were significantly better than lower grades (*p* = 0.017 and *p* = 0.018 respectively).

### 3.4 Analysis controlling for hearing loss level

To examine whether the observed age-related differences could be attributed to variations in hearing loss level among participants, a series of one-way Analyses of Covariance (ANCOVAs) were conducted. For the key dependent variables (Total Integrated Sequencing Score, Total Selection Score, Total Judgment Score), Grade was treated as a fixed factor and Hearing Loss Level (moderate, severe, profound) was included as a covariate.

The results indicated that the main effect of Grade remained significant for the Total Integrated Sequencing Score [*F*_(2, 75)_ = 15.01, *p* < 0.001] and the Total Selection Score [*F*_(2, 75)_ = 5.98, *p* = 0.004] after controlling for hearing loss level. The effect for the Total Judgment Score was also maintained [*F*_(2, 75)_ = 3.49, *p* = 0.036]. The covariate, Hearing Loss Level, was not a significant predictor in any of these models (*p* > 0.05). This confirms that the developmental trajectory related to age is robust across different levels of auditory deprivation within this cohort, and not primarily driven by differences in hearing loss level.

## 4 General discussion

### 4.1 Age-related characteristics and contextual factors in the development of temporal sequencing cognition

This study investigated the developmental characteristics of temporal cognition in hearing-impaired children through temporal sequencing, selection, and judgment tasks. The results demonstrated a clear age-related progression, with performance improving steadily from ages 11 to 17, and a particularly rapid growth phase observed around ages 14–15 for complex temporal tasks.

This trend aligns with the broader literature on cognitive development but must be interpreted within the specific context of our participant group. While Tong et al. (2012) found that temporal cognition improves with age in typically developing children due to increasing life experience and developing cognitive abilities, our findings reveal a delayed trajectory in hearing-impaired children. Research by Chen et al. (2012) identified age seven as a stabilization point for temporal sequencing cognition in typically developing children, whereas our study found hearing-impaired children experiencing a significant developmental phase during adolescence.

The finding that hearing-impaired adolescents exhibit a developmental progression in temporal sequencing that is both consistent with the typical pattern yet markedly delayed provides a nuanced contribution to the literature. This delay likely stems from multiple factors. First, the auditory and linguistic limitations associated with hearing loss (O’Neill, 2022; Ribeiro et al., 2022; Micheletti et al., 2020) create barriers to acquiring the abstract temporal concepts that hearing children absorb through incidental learning and language exposure. The significantly poorer performance on abstract time terms and culturally-specific festivals (which require explicit linguistic instruction) strongly supports this interpretation. Second, as noted by Oléron (1974), while deaf children demonstrate equivalent cognitive abilities to hearing peers during sensorimotor stages, they increasingly lag behind in more abstract cognitive domains requiring formal operational thinking. Crucially, our findings align with and extend research on other populations with special needs, which also shows that challenges in abstract temporal reasoning, such as sequencing cyclical events, are a common feature across various neurodevelopmental conditions (Titone, 1980).

Critically, these developmental patterns cannot be attributed to age alone. All participants in our study had received structured auditory rehabilitation and educational support within special education schools, yet still showed delayed development in abstract temporal reasoning. This underscores that despite intervention, the cumulative effects of auditory deprivation and reduced linguistic immersion continue to impact the development of higher-order temporal cognition into adolescence.

### 4.2 Developmental patterns and influencing factors of temporal sequencing cognition

This study revealed that hearing-impaired children perform better on short, concrete temporal sequencing tasks but struggle with long-term, integrated sequences, particularly those involving abstract concepts. These patterns reflect the interplay of multiple factors beyond mere chronological age.

The superior performance on concrete tasks aligns with Friedman’s (2002) spatial scaffolding hypothesis. Tasks like water flow sequencing provide visible spatial changes that offer concrete reference points, enabling accurate sequencing. Similarly, daily routines and human life stages offer distinct visual cues that align with lived experiences, facilitating temporal understanding.

In contrast, abstract temporal concepts (e.g., “last year,” “next month”) lack concrete referents and require linguistic and conceptual sophistication that hearing-impaired children may not have fully developed due to reduced access to temporal language in everyday interactions. This linguistic deprivation (Dang et al., 2008) compounds the inherent challenges of abstract temporal reasoning. Frostad’s (1999) observation that hearing-impaired children tend to rely on procedural rather than conceptual strategies is particularly relevant here, as our complex sequencing tasks required precisely the abstract conceptual thinking that proves challenging.

The future-oriented bias we observed warrants particular attention. While Wu et al. (2005) and Chen and Huang (2004) found hearing children excel at present-oriented tasks, our participants showed relative strength in future-oriented sequencing. This may reflect compensatory developmental pathways where visual–spatial strengths support forward-oriented planning more effectively than retrospective reconstruction of past sequences.

### 4.3 Integrated educational recommendations for supporting temporal cognition development

Our findings yield several implications for educational practice and intervention strategies, which must be considered within a comprehensive developmental framework.

#### 4.3.1 Early and sustained intervention beginning in infancy

We fully agree with Reviewer 2 that intervention for congenital hearing loss must begin immediately after diagnosis, ideally within the first year of life. Early fitting of hearing aids or cochlear implants and prompt auditory rehabilitation are absolutely critical for maximizing developmental outcomes (Yoshinaga-Itano et al., 2020). Our identification of ages 14–15 as a period of rapid development for complex temporal cognition should not be misinterpreted as suggesting intervention can wait until adolescence. Rather, it indicates that the cognitive foundations built through early intervention continue to enable growth into adolescence, and that sustained support during this period can capitalize on this developmental plasticity.

Early intervention should include rhythm perception activities and temporal processing training. Music therapy, rhythmic exercises, and technologies like LACE (Listening and Communication Enhancement) that integrate time-order judgment tasks can strengthen neural pathways for temporal processing (Fu and Galvin, 2007; Sweetow and Palmer, 2005).

#### 4.3.2 Environmentally rich and experientially grounded learning

Our results underscore that temporal cognition development is profoundly shaped by lived experience and structured learning opportunities. Educational approaches must leverage hearing-impaired children’s strengths in visual–spatial processing while systematically addressing their challenges with abstract concepts.

Vygotsky’s (1978) sociocultural theory emphasizes that structured social interactions and scaffolded language input are vital for bridging cognitive gaps. Implementational strategies should include:

Visual-temporal scaffolding using timelines, sequences, and spatial metaphors; Experiential learning that connects abstract temporal concepts to concrete experiences; Structured storytelling and narrative sequencing activities that explicitly teach temporal marker s; Family engagement in discussing temporal sequences in daily activities and stories.

#### 4.3.3 Integrated language and cognitive instruction across development

Language instruction must progressively integrate abstract temporal markers with concrete experiences, explicitly connecting linguistic forms to their temporal concepts. This is particularly crucial for hearing-impaired children, who cannot rely on incidental learning of temporal language.

Instruction should include: Explicit teaching of temporal vocabulary through multiple modalities (sign, print, visual representations); Sequencing activities that progress from concrete to abstract temporal concepts; Digital tools and apps designed to support temporal reasoning through interactive sequencing tasks; Curriculum integration that connects temporal concepts across subject areas.

In conclusion, the development of temporal cognition in hearing-impaired children represents a complex interaction between neurocognitive development, linguistic access, and educational experience. While age-related progression occurs, it is substantially mediated by the quality and timing of intervention, the richness of environmental support, and the strategic addressing of conceptual gaps through targeted instruction. A comprehensive approach that begins in infancy and continues through adolescence, capitalizing on periods of heightened plasticity while providing ongoing support, offers the optimal pathway for fostering temporal cognition development in this population.

### 4.4 Limitations and future directions

This study has several limitations. First, the lack of a hearing control group limits direct comparisons between hearing and hearing-impaired children. Future studies should include such a group to better isolate the effects of auditory deprivation. Second, although hearing loss level was included as a covariate and was not a significant factor, the diversity of audiological profiles within our sample underscores the heterogeneity of the hearing-impaired population. Future studies with larger samples could stratify participants by hearing loss level to explore more nuanced interactions. Third, the cross-sectional design prevents examination of individual developmental trajectories. Longitudinal studies would provide more insight into the developmental course of temporal cognition in hearing-impaired children. Fourth, while several tasks were adapted from established paradigms, the novel tasks designed for this study (Lotus Growth, Traditional Festivals) require further validation for reliability and construct validity in this population. Fifth, the research team did not include a clinical audiologist. Involving an audiologist in future research could further refine participant characterization and strengthen the interpretation of results. Finally, the educational recommendations derived from this study, while theoretically grounded, require empirical validation through intervention studies.

## 5 Conclusion

Hearing-impaired children’s temporal cognition develops progressively with age, exhibiting accelerated growth during early adolescence (14–15 years). Performance is superior in concrete, short-term tasks compared to abstract, long-term tasks, reflecting the impact of event salience and experiential familiarity. A future-oriented cognitive bias was observed, which may reflect unique compensatory mechanisms in temporal reasoning among hearing-impaired children.

These findings underscore the need for targeted interventions that align with deaf children’s cognitive profiles, emphasizing early rehabilitation, experiential enrichment, and multimodal language support to foster holistic temporal cognition development. Temporal cognition is not solely a function of age but is shaped by environmental stimuli, educational quality, and rehabilitation efforts. Future studies should examine how these factors interact with developmental trajectories.

## Data Availability

The raw data supporting the conclusions of this article will be made available by the authors, without undue reservation.
